# Connecting the DOTs on Cell Identity

**DOI:** 10.3389/fcell.2022.906713

**Published:** 2022-06-06

**Authors:** Coral K. Wille, Rupa Sridharan

**Affiliations:** ^1^ Wisconsin Institute for Discovery, University of Wisconsin-Madison, Madison, WI, United States; ^2^ Department of Cell and Regenerative Biology, University of Wisconsin-Madison, Madison, WI, United States

**Keywords:** H3K79, reprogramming, pluripotency, embryonic stem cell, DOT1l

## Abstract

DOT1-Like (DOT1L) is the sole methyltransferase of histone H3K79, a modification enriched mainly on the bodies of actively transcribing genes. DOT1L has been extensively studied in leukemia were some of the most frequent onco-fusion proteins contain portions of DOT1L associated factors that mislocalize H3K79 methylation and drive oncogenesis. However, the role of DOT1L in non-transformed, developmental contexts is less clear. Here we assess the known functional roles of DOT1L both *in vitro* cell culture and *in vivo* models of mammalian development. DOT1L is evicted during the 2-cell stage when cells are totipotent and massive epigenetic and transcriptional alterations occur. Embryonic stem cell lines that are derived from the blastocyst tolerate the loss of DOT1L, while the reduction of DOT1L protein levels or its catalytic activity greatly enhances somatic cell reprogramming to induced pluripotent stem cells. DOT1L knockout mice are embryonically lethal when organogenesis commences. We catalog the rapidly increasing studies of total and lineage specific knockout model systems that show that DOT1L is broadly required for differentiation. Reduced DOT1L activity is concomitant with increased developmental potential. Contrary to what would be expected of a modification that is associated with active transcription, loss of DOT1L activity results in more upregulated than downregulated genes. DOT1L also participates in various epigenetic networks that are both cell type and developmental stage specific. Taken together, the functions of DOT1L during development are pleiotropic and involve gene regulation at the locus specific and global levels.

## Introduction

Disruptor Of Telomeric silencing 1—Like (DOT1L) is an enigmatic histone methyltransferase that targets histone H3K79, a residue that is within the nucleosome core (Anderson et al., 2019; Valencia-Sánchez et al., 2019; Worden et al., 2019) and is conserved across several eukaryotes (Zhang et al., 2007; Nishida, 2009). Dot1 was first discovered in the yeast *Saccharomyces cerevisiae* where its deletion redistributes repressive silent information regulator proteins from the telomere leading to activation of expression at those locations (Singer et al., 1998). DOT1L is the only known H3K79 methyltransferase and performs all degrees—mono, di and tri—of methylation (van Leeuwen et al., 2002). Unless specified we will refer to all or any degree of H3K79 methylation as H3K79me. Unlike most other histone methylating enzymes (Black et al., 2012), it does not have a SET domain to perform catalysis (Min et al., 2003; Sawada et al., 2004). Additionally, DOT1L singularly targets H3K79 whereas the majority of other histone modifiers have multiple histone and non-histone targets. It is a distributive enzyme that falls off the substrate after each round of catalysis (Frederiks et al., 2008). Thus the degree (me1, me2, and me3) of H3K79me corresponds to local concentration of DOT1L. The degrees of H3K79 methylation also vary throughout the cell cycle [Reviewed here: (Kim et al., 2014)]. While there is some evidence of an H3K79 demethylase (Kang et al., 2018), methylation is thought to be reduced by nucleosome turnover (De Vos et al., 2011; Chory et al., 2019). Thus the majority of H3K79me in cells is controlled by DOT1L.

DOT1L is lowly expressed at both the mRNA and protein levels (Gillespie et al., 2020) and has many interacting partners that include transcriptional and chromatin remodeling proteins, and form mutually exclusive complexes (Mohan et al., 2010a; Park et al., 2010; Onder et al., 2012; Wu et al., 2021). In mammalian cell lines and *in vivo*, H3K79 methylation accumulates on the bodies of genes only after transcription commences (Steger et al., 2008) and is proportional to expression levels (Wang et al., 2008; Veloso et al., 2014). H3K79me2 is the only feature among various transcription related histone modifications and genomic features significantly correlated with a rapid transcriptional elongation rate (Duffy et al., 2018). H3K79 methylation also participates in extensive epigenetic crosstalk—its deposition is related to other modifications such as H2B ubiquitination from yeast to mammals (Briggs et al., 2002; Ng et al., 2002; Sun and Allis, 2002; Kim et al., 2005; Zhu et al., 2005; McGinty et al., 2008; Anderson et al., 2019; Valencia-Sánchez et al., 2019; Worden et al., 2019); and can affect accumulation of other histone modifications differentially at telomeres (Jones et al., 2008) and in actively transcribed genes (Deshpande et al., 2014; Chen et al., 2015). All degrees of H3K79me have been found at enhancers (Steger et al., 2008; Godfrey et al., 2019), and H3K79me2/3 maintain H3K27ac and enhancer activity in leukemia cell lines (Godfrey et al., 2019). In contrast to its connection with gene activity, H3K79me2 has also been detected at silencers (Pang and Snyder, 2020). Given these disparate properties, it has been difficult to pinpoint the function of DOT1L and H3K79 methylation. In this review we distill the current literature during cell fate determination and development focusing on vertebrate systems.

### DOT1L and H3K79 Methylation Dynamics in Early Embryogenesis

The fertilization of a lineage-committed oocyte triggers profound epigenetic and gene expression alterations to produce a totipotent zygote. Totipotency is a fleeting stage in development that gives rise to both the embryo and extra-embryonic tissues (Ishiuchi and Torres-Padilla, 2013). At the blastocyst stage, the inner cell mass and outer trophoectoderm demarcate future contributions to the embryo and extra-embryonic tissue, respectively. Immunofluorescence and chromatin immunoprecipitation sequencing (ChIP-seq) for low number of cells of epigenetic modifications after *in vitro* fertilization or parthenogenic activation allows for the observation of their relative abundance and spatiotemporal localization during this dramatic reorganization (Xia and Xie, 2020).

Oocytes, like other differentiated cells, are enriched for H3K79me2 and H3K79me3 (Ooga et al., 2008; Cao et al., 2015), express *Dot1l* mRNA, and have nuclear localization of DOT1L protein (Ooga et al., 2013). *Dot1l* depletion blocks metaphase of meiosis I and prevents loss of H3K27ac and H3K12ac during oocyte maturation (Wang et al., 2014), although a maternal specific knockout (KO) of DOT1L in oocytes does not affect the fertility of resultant progeny (Liao and Szabó, 2020). DOT1L is also important in post-mitotic spermatogenesis. Histones are highly modified with H3K79me immediately preceding the histone-to-protamine exchange during sperm differentiation by a *Dot1l* isoform containing an extended C-terminus, a process conserved in *Drosophila*, humans, mice, and rats (Dottermusch-Heidel et al., 2014b; 2014a). Reduction of DOT1L by depletion of male transcription factor SLY reduces H3K79me2 and retains histones in spermatozoa (Moretti et al., 2017). Although sperm are depleted for histones, approximately 20% contain H3K79me2; however, this modification is associated with low motility (La Spina et al., 2014) and thus sperm are likely not a significant source of H379me2 in the mouse embryo.

Two hours post fertilization in mice, H3K79me3 is undetectable and H3K79me2 is reduced, which then becomes largely depleted by 4 h when the female pronucleus is formed (Ooga et al., 2008). Similar H3K79me2 (Cao et al., 2015; Tao et al., 2017), but not H3K79me3 (Cao et al., 2015) kinetics are observed after fertilization of porcine oocytes. At this early timepoint, the embryo is still in G1 of the cell cycle and treatment with aphidicolin to halt DNA synthesis and S phase progression does not prevent loss of H3K79me2 (Ooga et al., 2008). Thus in the early hours post fertilization, the removal of H3K79me2/me3 could occur by histone exchange or the activity of an unknown demethylase. This lack of methylation is temporally regulated as H3K79me is depleted when somatic nuclei are transferred into an oocyte immediately after parthenogenetic activation, but not 10 h later (late 1-cell stage) (Ooga et al., 2008). Furthermore, the somatic nucleus fails to become reprogrammed when transferred at the later timepoint (Ooga et al., 2008).

**FIGURE 1:**
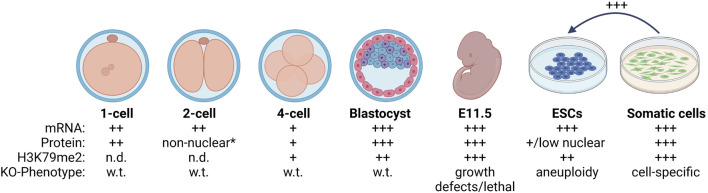
Summary of DOT1L in mammalian development. Oocytes express DOT1L (Ooga et al., 2013) and have detectable H3K79me2 (Ooga et al., 2008; Cao et al., 2015). H3K79me2 is quickly depleted after fertilization (Ooga et al., 2008) even though DOT1L is expressed and nuclear. At the 2-cell stage, DOT1L is evicted to the cytoplasm *except when H3K79me2 sharply increased during mitosis (Ooga et al., 2013; Yang et al., 2022). H3K79me2 begins to be detectable at the 4-cell stage when DOT1L mRNA and protein levels are low, and increases concurrent with DOT1L expression until the blastocyst stage (Ooga et al., 2008; Cao et al., 2015). DOT1L-KO is lethal beginning at E11.5 in the mouse (Jones et al., 2008; Feng et al., 2010; Liao and Szabó, 2020). *In vitro*, ESCs are globally depleted for H3K79me2 compared to somatic cells (Sridharan et al., 2013) and reducing DOT1L activity enhances reprogramming to induced pluripotent stem cells (Onder et al., 2012; Wille and Sridharan, 2022). Wild-type = w.t., and DOT1L knock-out = KO. The relative amount of DOT1L mRNA, protein, and H3K79me2 modification is indicated by: + or not detected = n.d. Created with BioRender.com.

At the 1-cell stage, *Dot1l* mRNA is found at comparable levels to that in oocytes, and the protein is detected in the nucleus (Ooga et al., 2013). DOT1L protein is evicted from the nucleus at the 2-cell stage, a low level is present at the 4-cell stage, and there is a large increase in nuclear DOT1L in the blastocyst (Ooga et al., 2013; Yang et al., 2022) (Figure 1). Mirroring the DOT1L dynamics, H3K79me2 remains undetectable until the 4-cell stage, except for a sharp increase at the M phase of the cell cycle, followed by a manifold increase in the blastocyst (Ooga et al., 2008; Cao et al., 2015). The 2-cell stage is when the major zygotic gene activation occurs to initiate transcription from the zygotic genome. The increase in H3K79 methylation from the 4-cell stage on concurs with the notion of transcription dependent deposition of this modification from other mammalian cell lines (Steger et al., 2008).

Together these data suggest independent, stage-specific mechanisms of H3K79me regulation during development from zygote to blastocyst (Figure 1): 1) Active removal of H3K79me post fertilization that is correlated with allowing development to proceed and occurs independent of DNA replication, 2) Accumulation of H3K79me beginning at the 4-cell stage (post zygotic gene activation) until the blastocyst stage *via* regulation of DOT1L localization and expression.

Absence of H3K79me2 post fertilization is evolutionarily conserved. In *Caenorhabditis elegans*, H3K79me2 is enriched in both sperm and oocytes and is rapidly removed at 1-cell stage (Samson et al., 2014). In *Xenopus tropicalis*, low H3K79me2 enrichment reflects levels of *dot1l* transcript. *Dot1l* is not expressed in eggs, begins to be activated at stage 8 during zygotic transcription in the mid-blastula transition (Wen et al., 2015), and increases until late metamorphosis (Matsuura et al., 2012). *X. tropicalis* Dot1l shares 65% identity with mouse DOT1L, and the methyltransferase domain is 99% identical (Matsuura et al., 2012). This pattern of rapid removal post fertilization (1-cell stage) and low enrichment until the blastocyst stage is exclusive to H3K79me2/3 in porcine (Cao et al., 2015) and mouse (Ooga et al., 2008) embryogenesis compared to other histone modifications [patterns reviewed here: (Lim et al., 2016)], and suggests a unique role in marking developmental time.

Moreover, H3K79me2 and H3K79me3 may have separate functions during development. During both oocyte (Ooga et al., 2008) and sperm maturation (Ontoso et al., 2014), H3K79me2 is found throughout chromosomes, while H3K79me3 is enriched at the pericentric heterochromatin regions. DOT1L activity maintains heterochromatin chromocenters after cells exit the 2C stage (see below) (Yang et al., 2022). In embryonic stem cells derived from mouse blastocysts, H3K79me2 at telomeres and major satellite repeats is important for epigenetic repression of pericentric heterochromatin (see below) (Jones et al., 2008). Thus, H3K79me2 and H3K79me3 may have important and distinct roles at active and repressed regions that are especially apparent during development when global chromatin reorganization establishes cell fate.

### DOT1L Function in Totipotency

Pericentromeric chromatin, which encompasses the major satellite repeats, forms a ring like structure in the totipotent 2-cell (2C) stage of development. After the 2C stage, pericentromeric chromatin forms chromocenters of colocalized centromeres that appear as nuclear puncta. This reorganization is dependent on transcription of major satellite DNA from the paternal chromosome [reviewed: (Lim et al., 2016)]. DOT1L protein is evicted from the nucleus at the 2C stage when pericentric heterochromatin forms a ring (Ooga et al., 2013; Yang et al., 2022). Forced nuclear maintenance of DOT1L at the 2C stage by deletion of its C-terminal domain after amino acid 416 (DOT1LΔC) results in precocious chromocenter formation (Ooga et al., 2013). This effect is observed even when the DOT1LΔC is catalytically mutated (Ooga et al., 2013) pointing to a protein rather than H3K79me level function for DOT1L in regulating chromocenter formation.

**FIGURE 2:**
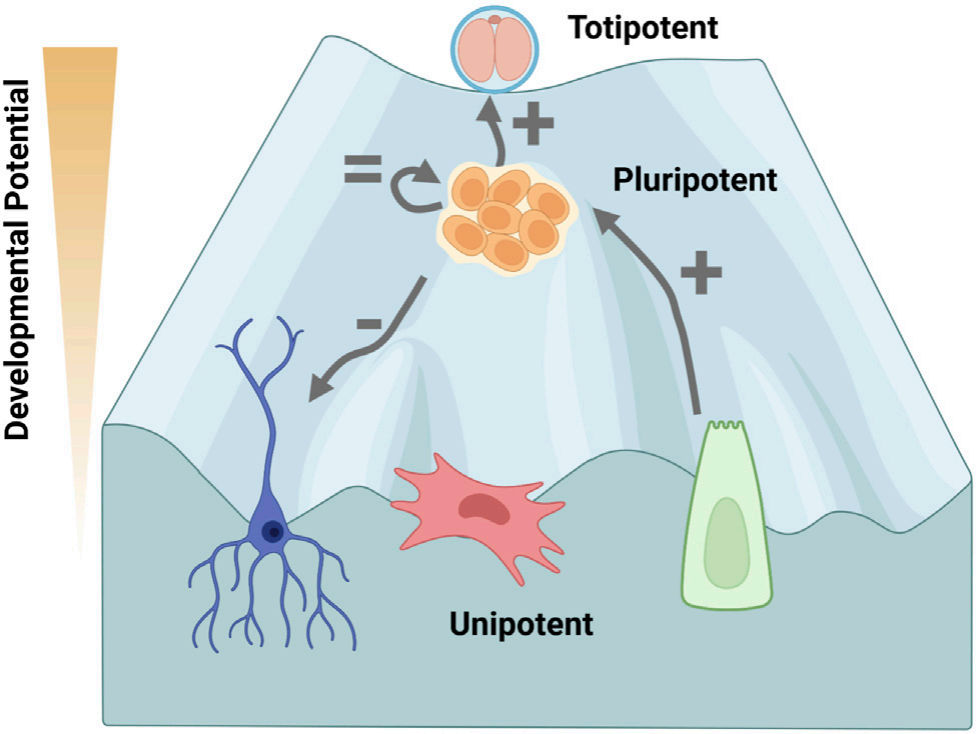
Decreased DOT1L activity is associated with increased developmental potential. The Waddington landscape envisions the process of cellular specification as a ball rolling down an incline, losing developmental potential as a new identity is gained. Cells cannot easily change lineage after cell fate decisions are specified, which become entrenched by epigenetic modifications. Totipotent cells in the 2-cell (2C) embryo, are on top of the landscape as they can differentiate into any cell type of the body or extra-embryonic tissue, whereas the inner cell mass of the pluripotent blastocyst differentiates into the embryo. DOT1L activity maintains chromocenter heterochromatin to oppose transition of pluripotent embryonic stem cells (ESCs) to a 2C-like cell state *in vitro* (Yang et al., 2022). DOT1L is not required for self-renewal of ESCs (Jones et al., 2008; Cao et al., 2020). DOT1L disruption inhibits differentiation *in vivo* and *in vitro* (Jones et al., 2008; Barry et al., 2009; Feng et al., 2010; Liao and Szabó, 2020). Conversely, inhibiting DOT1L greatly enhances reprogramming from unipotent somatic cells indicating H3K79me is a barrier for pluripotency acquisition (Onder et al., 2012; Wille and Sridharan, 2022). Thus, DOT1L activity is a determinate of developmental potential. The phenotype upon DOT1L disruption is depicted by: +, = , −. Created with BioRender.com.

*In vitro*, 2C-like cells (2CLC) spontaneously occur at about a frequency of 0.5% from embryonic stem cells (ESCs) (Macfarlan et al., 2012). ESCs are stem cells isolated from the inner mass of the blastocyst that can be propagated in culture to maintain pluripotent properties (Yu and Thomson, 2008). Although 2CLC express marker MERVL, their expression and epigenome still resemble ESCs (Ishiuchi et al., 2015; Hendrickson et al., 2017; Yang et al., 2022). Moreover, half of 2CLC contain chromocenters, and not ring structures (Yang et al., 2022). Genes that fail to be upregulated in 2CLC contain H3K79me2/3 suggesting that DOT1L may oppose totipotent expression (Figure 2). Pharmacological inhibition of DOT1L activity in 2CLC increases the appearance of the ringlike pericentric heterochromatin formation and enhances MERVL expression although H3K79me at these genes does not change (Yang et al., 2022). DOT1L inhibitor was also included in a cocktail to stably increase totipotent properties *in vitro* (Xu et al., 2022). We note that the DOT1L inhibitors used in these studies decrease chromatin association which may occur by directly opposing DOT1L function or by reducing transcription with the RNA polymerase II-associated DOT1L (Nassa et al., 2019; Wu et al., 2021). Thus, the effects of DOT1L inhibition could mimic protein depletion. Taken together these data suggest that totipotency requires absence of DOT1L to time chromocenter formation and potentially coordinate zygotic genome activation that occurs at this unique cell state.

### Regulation of DOT1L Activity in the Pluripotent Identity

ESCs can divide indefinitely while still being able to differentiate into all embryonic cell types. ESCs are isolated from the blastocyst where H3K79me2 is readily detected (Ooga et al., 2008), albeit still lower compared to differentiated cell types like keratinocytes (Wille and Sridharan, 2022) (Figure 1). In fact, H3K79me2 is the most reduced histone lysine methylation in ESCs compared to mouse embryonic fibroblasts (MEFs) by unbiased mass spectrometry (Sridharan et al., 2013). H3K79me2 is the most elevated modification assessed upon *in vitro* neuronal differentiation of ESCs (Ferrari et al., 2020) and increases during T cell maturation (Scheer et al., 2020). However, unlike 2C cells, both ESCs and differentiated cells share the chromocenter structure of pericentric heterochromatin, hence the requirement for ESCs to maintain low H3K79me is likely to be different than in 2C cells. Even with the uniquely low H3K79me2 enrichment, *Dot1l* mRNA is expressed at a comparable level in MEFs and ESCs (Wille and Sridharan, 2022). Like early embryogenesis, DOT1L is regulated at the level of protein localization. High activity of the cell cycle regulators CDK1 and CDK2 in ESCs, which is not observed in somatic cells, results in DOT1L phosphorylation at S1105 and nuclear eviction (Michowski et al., 2020). CDK1 chemical inhibition allows for DOT1L nuclear translocation, increases H3K79me2, and enhances endoderm gene expression during differentiation (Michowski et al., 2020). Of note, the DOT1LΔC truncation that maintains DOT1L in the nucleus at the 2C stage (Ooga et al., 2013) removes the CDK1 target residue. Thus, the nuclear eviction of DOT1L at the 2C stage could be a result of CDK1 activity.

DOT1L activity is not required for self-renewal of ESCs since DOT1L-KO ESCs can be isolated (Jones et al., 2008) and propagated (Jones et al., 2008; Cao et al., 2020) (Figure 2). Although DOT1L-KO ESCs appear phenotypically wild-type (Cao et al., 2020), they have a 2-fold increase in apoptosis and increased rates of G2 cell cycle arrest (Jones et al., 2008). DOT1L-KO results in elongation of telomeres *via* the alternative lengthening of telomeres (ALT) pathway, and aneuploidy suggesting disruption of chromosomal segregation (Jones et al., 2008). Chemical inhibition of DOT1L has also been shown to decrease fidelity of mitosis in transformed human cell lines (Guppy and McManus, 2015). In the case of *Dot1l* depletion using shRNA, proliferation is negligibly affected in ESCs, but is reduced during retinoic acid-mediated and embryoid body differentiation (Barry et al., 2009). Differentiation of knockdown ESCs increases the number of cells in G2 arrest, the number of apoptotic cells, and the proportion with aneuploidy (Barry et al., 2009) suggesting that H3K79me2 is vital for somatic cells. Functionally, *Dot1l*-depleted ESCs can form teratomas, but appear more glandular/epithelial and retain *Oct4* expression suggesting a failure to differentiate (Barry et al., 2009). Thus, despite low-level mitotic arrest, pluripotent cells are tolerant, and even actively promote, low levels of DOT1L activity (Figure 2).

### Pluripotency Acquisition

A direct link between low H3K79me and pluripotency has been established using somatic cell reprogramming to induced pluripotent stem cells (iPSCs). In this process, a small set of proteins - commonly the Yamanaka factors OCT4, SOX2, c-MYC, and KLF4 - are introduced into somatic cells to obtain iPSCs, which are functionally equivalent to ESCs, at a low efficiency (∼0.1%–5%). In 2012, *Dot1l* was identified from an shRNA screen of epigenetic modifiers as one of the top barriers of human fibroblast reprogramming (Onder et al., 2012). Methylation of H3K79, and not DOT1L protein itself, inhibits pluripotency acquisition as EPZ004777, a first generation DOT1L catalytic inhibitor (Daigle et al., 2011), comparably enhanced reprogramming (Onder et al., 2012). DOT1L inhibition did not alter cellular proliferation (Onder et al., 2012; Wille and Sridharan, 2022) or expression of reprogramming transgenes (Onder et al., 2012) demonstrating a role beyond augmentation of reprogramming-capable cells.

DOT1L depletion and/or catalytic inhibition also enhances mouse reprogramming of tail tip fibroblasts (Onder et al., 2012), peripheral blood cells (Onder et al., 2012), neural stem cells (Jackson et al., 2016), keratinocytes (Wille and Sridharan, 2022), and MEFs (Onder et al., 2012; Wille and Sridharan, 2022) (Figure 2). DOT1L inhibition enhances pluripotency throughout the process, but acts most potently at the early stages of low efficiency human fibroblast reprogramming, (days 0–7) (Onder et al., 2012; Kim et al., 2021), and at the mid-point (days 2–4) of MEF reprogramming when using a more efficient inducible system (Wille and Sridharan, 2022).

The second generation DOT1L inhibitor, SGC0946 (which is 10-fold more potent than EPZ004777) (Yu et al., 2012; Kaniskan et al., 2018), was identified in combination with ascorbic acid and signaling inhibitors for super-efficient ∼45% MEF reprogramming (Tran et al., 2019). DOT1L inhibitors have been included in the cocktail for completely chemical reprogramming in the absence of transgenes of MEFs (Zhao et al., 2018), mouse neural stem cells (Ye et al., 2016), and even human embryonic fibroblasts to iPSCs (Guan et al., 2022). Additionally, EPZ004777 increased blastocyst rate of porcine cell somatic cell nuclear transfer (SCNT) (Tao et al., 2017). EPZ004777 in combination with a WNT inhibitor was insufficient to reprogram notoriously refractory bovine mesenchymal stem cells to iPSCs, but could induce morphological changes (Su et al., 2021). Thus overwhelming evidence indicates that H3K79me is a barrier for pluripotency acquisition across cell types and species (Figure 2).

### DOT1L Transcriptional Function in Pluripotency and *in Vitro* Differentiation

H3K79me2 is located on the bodies of active genes, and its presence is positively correlated with gene expression (Steger et al., 2008; Wang et al., 2008; Veloso et al., 2014). DOT1L directly interacts with the phosphorylated carboxy-terminal domain of RNA polymerase II (RNAPII) (Kim et al., 2012). However, much of the functional studies have been performed in leukemias under the context of onco-fusion proteins [reviewed: (Mohan et al., 2010b; Takahashi and Yokoyama, 2020)]. Therefore, pluripotency and reprogramming are ideal platforms to interrogate the role of DOT1L in non-transformed cells, in an environment of dynamic regulation of H3K79me2. A comprehensive GRO-Seq analysis that uncoupled transcriptional initiation and elongation in ESCs revealed that H3K79me2 and exon density were the two features most significantly correlated to rapid transcriptional elongation rate (Jonkers et al., 2014). Surprisingly, *Dot1l* deletion and catalytic inactivation result in exceedingly few transcriptional changes in ESCs (Barry et al., 2009; Cao et al., 2020) even though transcriptional rate partially corresponds to steady-state expression (Jonkers et al., 2014). Significantly more genes are differentially expressed (DE) upon induction of neuronal differentiation from ESCs (Barry et al., 2009; Cao et al., 2020). Disruption of the catalytic function of DOT1L yields even fewer DE genes during differentiation, and therefore the protein may function beyond methylating H3K79 (Cao et al., 2020). In ESCs, DOT1L-KO does not affect RNAPII genic distribution or initiation frequency (Cao et al., 2020). However, in combination with the added stress of the super elongation complex (SEC) inhibitor KL-2, transcriptional elongation is lessened by DOT1L-KO, but not catalytic mutation (Cao et al., 2020). RNAPII signal is decreased at the transcription termination site, especially at highly expressed genes, indicating a potential role in mRNA 3’ end processing (Cao et al., 2020). When RNAPII pause release and elongation are disrupted by depleting *Pfh5a* with shRNA, H3K79me2 is reduced and self renewal of ESCs is diminished (Strikoudis et al., 2016, 2017).

**FIGURE 3:**
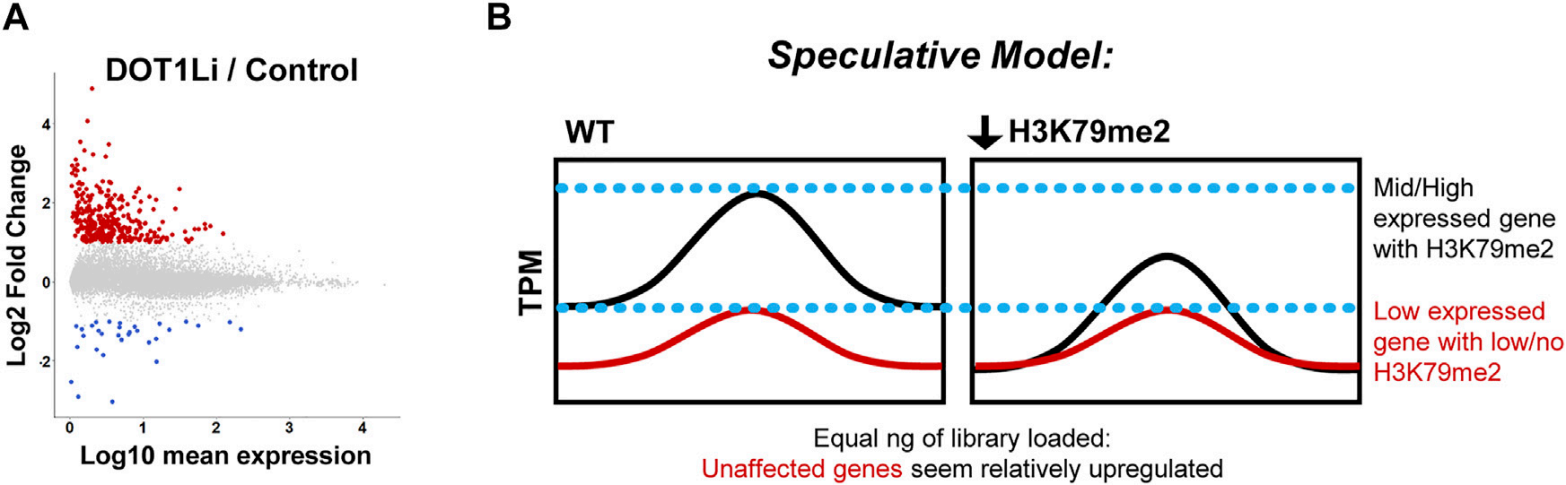
Transcriptional effects of DOT1L disruption. **(A)**. Differentially expressed genes upon DOT1L chemical inhibition are mostly lowly expressed and upregulated, displayed as Log10 mean expression (TPM) versus Log2 fold change in DOT1Li relative to Control treatment. At least 2-fold upregulated = red, downregulated = blue. Note that the exact number and scale of differentially expressed genes are specific to Wille and Sridharan, 2022 from which the figure was adapted. **(B)**. Non-normalized RNA-seq artifact speculative model: If H3K79me2 is a global positive regulator of transcription, its removal could uniformly lower transcription of modified genes. Thus, the unchanged, non-H3K79me2 modified genes seem more abundant when equal amounts of library are interrogated.

Even with the dramatic increase in pluripotency acquisition, RNA-seq during both human (Onder et al., 2012) and mouse (Wille and Sridharan, 2022) reprogramming reveals very few transcriptional alterations, even though H3K79me2 is enriched at approximately 10,000 genes in reprogramming MEFs (Wille and Sridharan, 2022). More genes are affected by DOT1L chemical inhibition compared to shRNA depletion and very few overlap (Onder et al., 2012). This may be because of a more complete reduction of DOT1L activity with the inhibitor, but off-target effects cannot be discounted. Although H3K79me2 is associated with gene activity, many more genes are upregulated than downregulated by DOT1L inhibition during reprogramming (Onder et al., 2012; Wille and Sridharan, 2022) (Figure 3A). This is opposite of what would be expected upon removal of a positive transcriptional regulator. A bias to increased gene expression by DOT1L disruption has been observed in numerous studies for example in mESCs (Barry et al., 2009; Cao et al., 2020; Ferrari et al., 2020) and during differentiation (Ho et al., 2013; Franz et al., 2019; Kealy et al., 2020; Kwesi-Maliepaard et al., 2020; Scheer et al., 2020; Aslam et al., 2021; Sutter et al., 2021; Borosha et al., 2022). Commonly, the genes that are upregulated are lowly expressed (Ferrari et al., 2020; Kwesi-Maliepaard et al., 2020; Wille and Sridharan, 2022), albeit total expression may not imply functional importance; and have low/no H3K79me2 (Ho et al., 2013; Franz et al., 2019; Ferrari et al., 2020; Kwesi-Maliepaard et al., 2020; Aslam et al., 2021; Wille and Sridharan, 2022) (Figure 3A). Some reports have shown that in contrast to the more numerous upregulated genes upon DOT1L disruption, downregulated genes tend to have H3K79me enrichment and/or higher levels of expression (Franz et al., 2019; Ferrari et al., 2020; Kwesi-Maliepaard et al., 2020; Scheer et al., 2020; Aslam et al., 2021; Wille and Sridharan, 2022). Thus, while these downregulated genes are lesser in number, they could be the true, direct targets of DOT1L. Single cell (sc)-RNA-seq analysis of high efficiency MEF reprogramming when DOT1L inhibition is combined with other epigenetic and signaling molecules revealed that SGC0956 is more important for activating the pluripotency gene regulatory network rather than suppressing somatic gene expression (Tran et al., 2019).

Although H3K79me2 is thought to be positive regulator of transcription, these cofounding, reproducible transcriptome-level effects could be due to: 1) Loss of H3K79me and gene expression of transcriptional repressor(s), 2) A consistent global reduction of transcription at all H3K79me-enriched genes such that genes without modification that do not change in expression appear elevated due to artifacts of bulk RNA-Seq (Figure 3B), 3) A negative transcriptional function of H3K79me, a phenomenon observed in *C. elegans* (Cecere et al., 2013), or 4) Alterations in an epigenetic network that do not lead to immediate transcriptional changes but establish special chromatin states such as poising (see below). To distinguish these possibilities, future studies of DOT1L transcriptional effects could employ spike-in controls at the level of cell number rather than RNA amount. A similar phenomenon occurs with *c-Myc* deletion such that transcriptional changes are obscured when measured at the non-normalized bulk RNA-seq level (Lin et al., 2012; Nie et al., 2012). In fact, *Dot1l* depletion can substitute for *c-Myc* in the Yamanaka reprogramming combination (Onder et al., 2012) indicating a potential overlap between their functions in the acquisition of pluripotency. However, notably, leukemia cells treated with a DOT1L chemical inhibitor did not have a significant difference in the global transcriptome when multiple spike-ins at the level of cell number were employed (Richter et al., 2021).

To challenge the first possibility, a comprehensive screen of genes directly downregulated by DOT1L inhibition during reprogramming identified the potential target *Nfix*, both a transcriptional activator and repressor (Messina et al., 2010), but its depletion was insufficient to substitute for loss of H3K79me (Wille and Sridharan, 2022). In contrast, in human reprogramming, expression of DOT1L-repressed genes NANOG and LIN28A supplant for DOT1L depletion, and expression of DOT1L-activated mesenchymal genes block DOT1L inhibited (DOT1Li)-mediated reprogramming (Onder et al., 2012). These discrepancies in the causes of DOT1L effects on maintenance of cell identity may reflect species or reprogramming efficiency related differences in the two studies.

### H3K79me Crosstalk With Chromatin Modifications

#### Reprogramming

**FIGURE 4:**
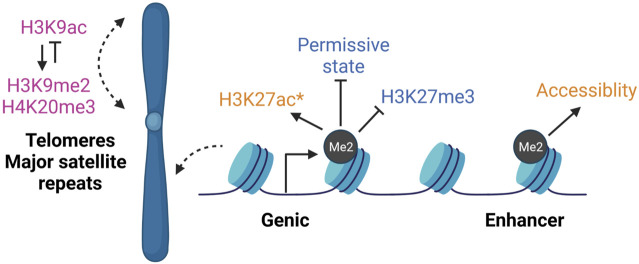
DOT1L epigenetic cross-talk. DOT1L inhibition largely does not affect gene expression even though H3K79me is enriched on the bodies of thousands of actively transcribed genes. However, H3K79me2 participates in various epigenetic networks that may not have immediate transcription effects. In DOT1L-KO ESCs (pink), repetitive regions like telomeres and major satellite repeats lose H3K9me2 and H3K20me3, and gain H3K9ac (Jones et al., 2008). During reprogramming (blue), DOT1L inhibition promotes a permissive state that allows OCT family members bind (Kim et al., 2021). In contrast, H3K79me2 opposes spread of H3K27me3 into active genes (Onder et al., 2012). During neuronal differentiation from ESCs (orange), DOT1Li reduces enhancer accessibility and decreases H3K27ac (*at select downregulated genes) (Ferrari et al., 2020). Thus, H3K79me2 has been reported to modulate both activating and repressive modifications, indicating both cell type- and local chromatin-specific effects, rather than a single global cross-talk mechanism. Created with BioRender.com.

Given that the loss of H3K79me has few transcriptional effects, it remains possible that H3K79me2 may alter other histone modifications to set up a cell type specific epigenetic platform. For example, during human reprogramming, among 348 genes that have reduced H3K79me2 levels compared to the starting somatic fibroblasts, 12 are decreased in expression during reprogramming (Onder et al., 2012). The use of DOT1Li modestly reduces the expression and increases the repressive modification H3K27me3 at 4 target mesenchymal genes that are decorated with H3K79me2 in fibroblasts (Onder et al., 2012). This immediate transcriptional decrease could be due to the spread of H3K27me3 at specific locations that marks them for further complete shutdown at later stages of reprogramming (Figure 4). Spread of H3K27me3 upon H3K79me2/3 reduction has been observed in leukemia (Deshpande et al., 2014). In contrast, a second study in leukemia using an internally calibrated ChIP-seq method found that downregulated genes had a confounding loss of H3K27me3 when H3K79me2 was reduced using the lower concentration of the DOT1Li EPZ5676 (Richter et al., 2021). Furthermore, in lymphocytes, DOT1L-KO de-repressed genes that are normally shut-off by H3K27me3, and slightly decreased the expression of the H3K27 tri-methyltransferase EZH2 (Kwesi-Maliepaard et al., 2020; Aslam et al., 2021). Thus, there have been both positive and negative connections of H3K79me2 and H3K27me3 in blood cells, an association not yet fully explored in pluripotency.

DOT1Li also enhances human reprogramming in combination with the inhibitors that block the bromodomains of histone acetyltransferases EP300 and CBP but not their catalytic activity (Ebrahimi et al., 2019). Bromodomains read histone lysine acetylation to cause downstream effects.

Beyond these site-specific effects, out of 165 tested compounds, the three commercial DOT1Li (SGC0946, EPZ5676, and EPZ004777) (Kaniskan et al., 2018) most potently create a permissive epigenetic state allowing other OCT family members to substitute for OCT4 during human reprogramming (Kim et al., 2021) (Figure 4). SGC0946 increases OCT7 binding to OCT4 targets without directly increasing the transcriptional activity of OCT family members (Kim et al., 2021). The exact mechanism of how this permissive state is generated remains unknown.

#### Pluripotency

Unlike in reprogramming, DOT1Li treatment upregulated genes are already marked by H3K27me3 in ESCs (Ferrari et al., 2020), similar to the observations in lymphocytes (Kwesi-Maliepaard et al., 2020; Aslam et al., 2021). Hidden Markov modeling can be used to identify patterns of co-occurring chromatin modifications called “states” (Ernst and Kellis, 2012). Chromatin state signatures of genes upregulated upon DOT1L inhibition in ESCs were enriched for H3K27me3 alone or with H3K4me3 and downregulated genes had a copresence of H3K4me3/H3K27ac (Ferrari et al., 2020). However mathematical modeling indicated that a particular chromatin state could not sufficiently explain differential expression (Ferrari et al., 2020). The DOT1L chemical inhibitor EPZ5676 largely did not affect H3K27ac deposition or ATAC-seq accessible regions with the exception of select enhancers in ESCs (Ferrari et al., 2020). However, during neuronal differentiation from ESCs, DOT1Li decreased H3K27ac at select downregulated genes, and reduced enhancer accessibility and binding by SOX2 (Ferrari et al., 2020) (Figure 4). Correspondingly, DOT1L-mediated H3K79me2/3 was required to preserve accessibility and H3K27ac of intergenic enhancers in leukemia cell lines (Godfrey et al., 2019). Thus, there may be an H3K79me—H3K27me/ac network unique to pluripotency.

In DOT1L-KO ESCs, heterochromatin enriched telomeres and major satellite repeats gain H3K9ac and lose repressive modifications H3K9me2 and H4K20me3 (Jones et al., 2008) (Figure 4). Epigenetic de-repression may promote telomere elongation found in DOT1L-KO ESCs (Jones et al., 2008). In direct contrast, in leukemia, loss of H3K79me upon DOT1Li reduced H3K9ac and promoted H3K9me2 at target genic loci *via* a complex containing the deacetylase SIRT1 and H3K9 methyltransferase SUV39H1 (Chen et al., 2015). Thus loss of H3K79me2 may result in local histone modification effects on subsets of single copy genes, as well as have a unique role at repeats, rather than a single global cross-talk mechanism, which may be further specific to cellular environment.

### Development and Differentiation

Although H3K79me is a highly dynamic modification during fertilization and early development, its function is unclear. Three independent constitutive DOT1L-KO mice, using different strategies, have been generated and are embryonic lethal from E10.5-E13.5 (Table 1). Thus, the global gain of H3K79me2 that begins at the blastocyst stage is not required until later in development. In two DOT1L-KOs, embryos have growth retardation by E9.5 and 100% lethality by day E11.5 (Jones et al., 2008; Liao and Szabó, 2020). Other phenotypes include cardiac dilation, areas of apoptosis, and stunted tails (Jones et al., 2008). In the third DOT1L-KO model, some embryos survive beyond E11.5 (Feng et al., 2010). Characterization of extra-embryonic tissues reveal defects in angiogenesis such as disorganized and underdeveloped yolk sac vasculature (Jones et al., 2008; Feng et al., 2010). Feng et al. observed severe anemia with a reduction in blood cells due to increased apoptosis and cell cycle block of erythroid progenitors. The less differentiated hematopoietic progenitors in the yolk sac are unaffected by DOT1L-KO. Vessel disorganization also occurs in the fetal brain at 10.5 upon DOT1L-KO in endothelial cells demonstrating that DOT1L is important for development beyond the extra-embryonic tissue (Yoo et al., 2020).

**TABLE 1:** Complete DOT1L-KO studies.

Complete-KO strategy	H3K79me	Phenotype
Exon 5–6 LoxP (catalytic domain, SAM binding motif)	Mass Spectrometry	E8.5: Wild-type E9.5: Small, enlarged heart, stunted tail, 15% arrest, focal apoptosis, abnormal vascular morphology of the yolk sac E10:5: Developmental arrest, cardiac dilation, reduced Mendelian ratio **E11.5: 100% lethality** Jones et al. (2008)
Exon 13 Gene trap (nucleosome binding region)	H3K79me1/2/3 immunoblot	E9.5: Wild-type E10.5: Small, absence of red blood cells, abnormal vascular morphology of the yolk sac E11.5: Reduced Mendelian ratio **E13.5: 100% lethality** Feng et al. (2010)
Exon2 LoxP (out of frame)		E9.5: Retarded E10.5: Retarded **E11.5: 100% lethality** Liao and Szabó. (2020)
Dot1l Asn241Ala (Exon 9)	H3K79me2 immunoblot in ESCs and E10.5 embryos	E10.5: Small, normal Mendelian ratio E11.5: 10% embryos dead **E13.5: 100% lethality** Malcom et al. (2022)

E, embryonic day.

A catalytically inactive mutant (DOT1L-N241A) mouse model of DOT1L histone methyltransferase activity (HMT) reveals specific effects on blood cell formation (Malcom et al., 2022). Structural studies have shown that N241 is a conserved amino acid at a channel leading to the SAM binding pocket that is required for catalytic activity without affecting protein folding (Min et al., 2003). Although the HMT-mutation is lethal by E13.5, embryos are not overtly anemic as the extra-embryonic yolk sacs contain blood and are vascularized at E10.5 (Malcom et al., 2022) in contrast to the DOT1L-KO model (Feng et al., 2010). DOT1L catalytic activity is required for definitive (myeloid and mixed lineage colonies), but not primitive, hematopoiesis when assessed *ex vivo* (Malcom et al., 2022). Thus, DOT1L catalytic activity, not protein integrity, is specifically required for the late stages of embryonic hematopoiesis. Furthermore, in a conditional DOT1L-KO when embryos were allowed to develop until day 9.5 and *Dot1l* was knocked out using tamoxifen induced CRE-ER, a high dose (1.25 mg/25 g) was completely lethal by E14.5-E15.5 (Yoo et al., 2020). A lower dose of tamoxifen caused hypoplastic mesenteric lymphatics in half the embryos by E17.5 (Yoo et al., 2020). Therefore, DOT1L is required throughout organogenesis.

**FIGURE 5:**
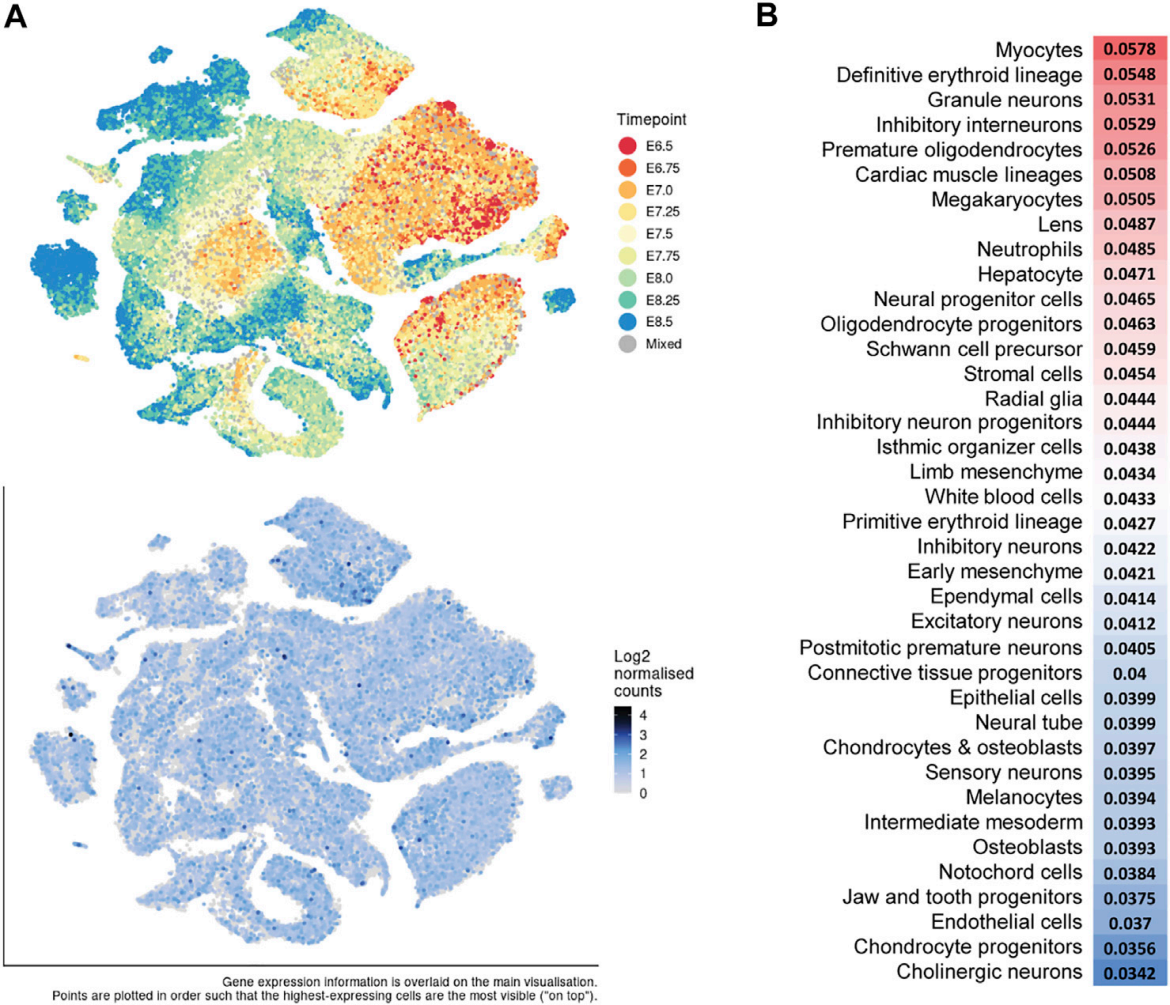
*Dot1l* expression during mouse gastrulation and organogenesis. **(A)** t-distributed stochastic neighbor embedding (t-SNE) plots of all single cells (E6.5–E8.5) of a mouse gastrulation atlas. Top: Cells labeled by developmental timepoint. Bottom: *Dot1l* expression (blue) analyzed from Pijuan-Sala et al., 2019: https://marionilab.cruk.cam.ac.uk/MouseGastrulation2018/. **(B)**. Relative *Dot1l* expression across all timepoints (E9.5–E13.5) per cell type analyzed from a mouse organogenesis sc-RNA-seq atlas (Cao et al., 2019). Red = higher expression and blue = lower expression. http://atlas.gs.washington.edu/mouse-rna.

Interestingly, *Dot1l* reporter gene expression is uniform at the earliest timepoint assessed, E7.5, but is elevated in the extra-embryonic tissues of the visceral endoderm and mesoderm of the yolk sac, and in primitive erythrocytes by E9.5 (Jones et al., 2008). It is also increased at E9.5 in following embryonic tissues: the optic vesicle, the first branchial arch, the limb buds, the heart, the otic pit, and the neural ectoderm. We analyzed *Dot1l* expression from two recently published single-cell transcriptional atlases during mouse gastrulation (Pijuan-Sala et al., 2019) and organogenesis (Cao et al., 2019) and found that it is expressed across all developing lineages from E6.5 to E8.5 and from E9.5–E13.5 (Figures 5A,B). During organogenesis, there is slightly higher relative expression in myocytes and definitive erythroid lineage (Cao et al., 2019) (Figure 5B). Thus, developmental dysregulation coincides in part with the timing and the location of differential *Dot1l* expression. The broad expression (Briggs et al., 2018), and later requirement for DOT1L beyond the beginning of H3K79me, is conserved in *Xenopus* (Wen et al., 2015). Deletion of d*ot1l* with TALEN mRNA in fertilized eggs results in smaller tadpoles that die from day 8 to day 20 post-fertilization (Wen et al., 2015), a time window corresponding to mammalian organogenesis (Theiler stage 17).

Differentiation of almost all organ systems and a 100-fold expansion in the number of cells occurs from E9.5–E13.5 (Cao et al., 2019), when DOT1L-KO mouse embryos rapidly become non-viable. There is very little change in expression and transcript diversity throughout gastrulation (E6.25–E7.5), but a burst of transcript diversity occurs at the beginning of organogenesis at E8.0 (Mitiku and Baker, 2007). At this timepoint, expression switches from pluripotency enriched genesets to differentiated sets such as cardiac and vascular markers (Mitiku and Baker, 2007). Therefore, DOT1L is dispensable until exit from gastrulation but is required during transcript diversification and development of differentiated tissues.

**FIGURE 6:**
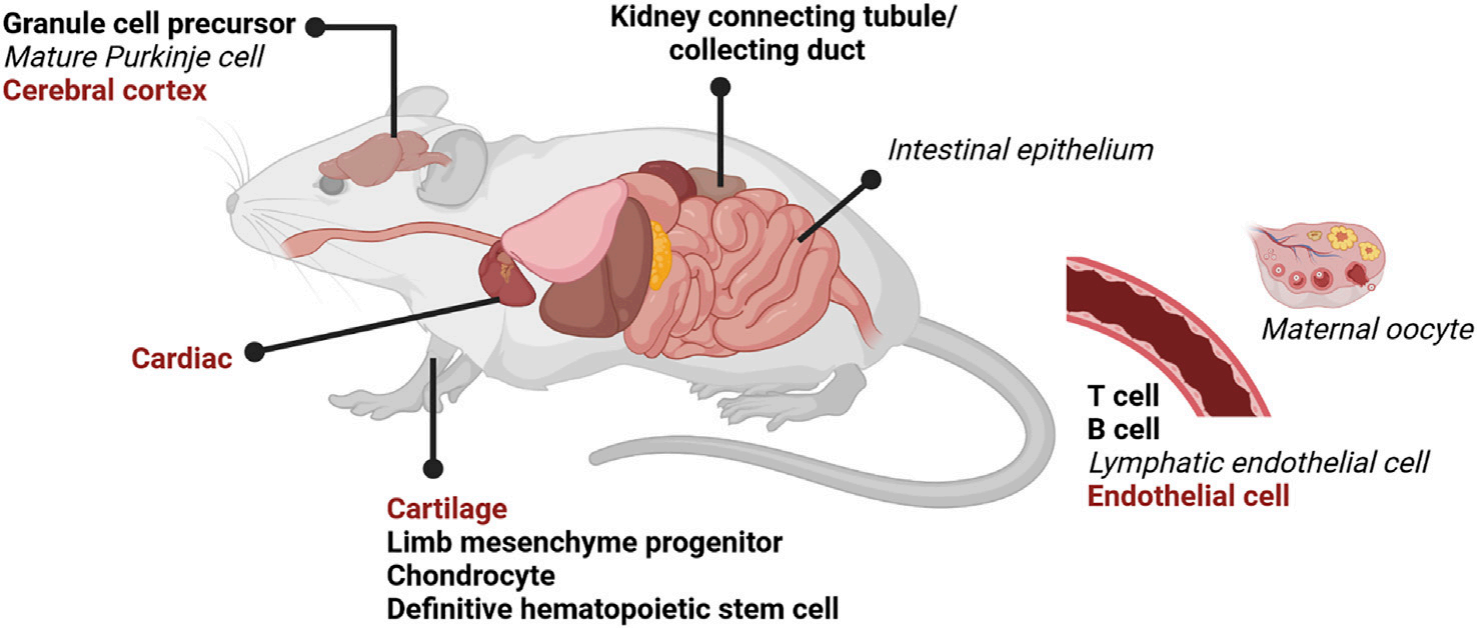
DOT1L lineage-specific knock-out (KO) mouse models. Numerous lineage DOT1L-KO mouse models using tissue-specific CRE drivers have recently been reported (Table 2). Total DOT1L-KO is embryonic lethal around E11.5 (Table 1). Tissue-specific KOs have various phenotypes, but the overwhelming majority have decreased or impaired differentiation. Perinatal lethality is indicated in red bold, disrupted differentiation in black bold, and no phenotype in black italics. Created with BioRender.com.

Total DOT1L-KO is likely lethal from severe anemia (Feng et al., 2010; Malcom et al., 2022), and therefore blood differentiation could be particularly sensitive to loss of H3K79me2, or the requirement for DOT1L may be revealed first in this early differentiating population. Lineage-specific DOT1L-KO during mouse development has recently been explored in numerous tissues with various CRE drivers (Table 2) (Summarized in Figure 6). DOT1L-KO in cardiac tissue (Nguyen et al., 2011), cerebral cortex (Franz et al., 2019), endothelium (Yoo et al., 2020), and cartilage (Jo et al., 2020) results in perinatal lethality. DOT1L-KO in the cerebellum does not affect viability but does disrupt tissue organization and cause motor defects (Bovio et al., 2019). In many cases, DOT1L is required for tissue organization, yet the direct contribution to differentiation is lineage specific. Targeted DOT1L-KO in endothelial cells results in lymphatic aplasia whereas overexpression promotes hyperplastic lymphatics (Yoo et al., 2020). DOT1L-KO maintains fetal gene expression and cell proliferation in the heart suggesting that cells cannot terminally differentiate without H3K79me (Nguyen et al., 2011). In one study, B cells are stuck at the pro-B stage, yet have a more differentiated, plasma cell-like transcriptional program (Aslam et al., 2021); yet in a mature B cell KO, germinal centers and plasma cells are impaired (Kealy et al., 2020). In contrast to these phenotypes, DOT1L-KO in T cells (Kwesi-Maliepaard et al., 2020) and the cerebral cortex (Franz et al., 2019) disrupts function by precocious differentiation of the progenitor pool. Thus, various KO models (Figure 6) demonstrate that DOT1L is dispensable during early stages of lineage specification, but broadly required for somatic differentiation (Figure 2).

**TABLE 2:** Tissue specific DOT1L-KO studies during development or differentiation.

Tissue	KO strategy	H3K79me	Phenotype
Maternal oocyte	*Zp3*-Cre, *Dot1l* Exon2 LoxP	H3K79me1/2 IF of oocytes	No affect on development or fertility of F1 pups. No affect on establishment imprinting in oocyte or maintenance in embryo. Liao and Szabó. (2020)
Cartilage	*Col2a1*-Cre, *Dot1l* Exon2 LoxP		Severely reduced Mendelian ratio of live births (8/58 expected). Only one survived to adulthood but with growth retardation. P2 pups had accelerated ossification and advanced mineralization. Aberrant *Col10a1* expression in epiphyseal chondrocytes. Jo et al. (2020)
Limb mesenchyme progenitor	*Prrx1*-Cre, *Dot1l* Exon5 LoxP	H3K79me2 IB of chondrocytes	Long bone shortening at birth, structural forelimb abnormalities in neonates, forelimb dislocations, fibula thickening, disorganized growth plate at 3 weeks, poor locomotion, reduced extracellular matrix production. Sutter et al. (2021)
Chondrocyte	*Col2*-cre, *Dot1l* Exon2 LoxP		Severe growth retardation, disorganized prehypertropic zone of growth plates, and increased WNT pathway activation. Monteagudo et al. (2017)
Intestinal epithelium	*Villin*-CreER, *Dot1L* Exon5 LoxP	H3K79me2 IHC of intestinal crypts	Induced deletion with tamoxifen at 4 weeks. 3 weeks to 4 months post deletion: No weight loss or malnourishment. Normal intentional morphology. Increased crypt apoptosis. Very few transcriptional alterations. Ho et al. (2013)
Granule cell precursor (cerebellum)	*Atoh1*-Cre *Dot1l* Exon2 LoxP	H3K79me1/2/3 IF of cerebellum	Small cerebellum with motor deficiencies at 9 weeks. At P3: Thin external granular layer and disorganized internal granular layer of cerebellum. Fewer dividing and differentiating progenitor cells. Bovio et al. (2019)
Mature Purkinje cell	*Pcp2*-Cre *Dot1l* Exon2 LoxP	H3K79me1/2/3 IF of cerebellum	No obvious phenotype. Bovio et al. (2019)
Cerebral cortex	*Foxg1*-Cre, *Emx1*-Cre, *Dot1l* LoxP		Lethality minutes after birth, microcephaly of ventral and dorsal structures at P0, depletion of neural progenitor cells through premature differentiation into deep layer neurons at E12.5–E14.5. Franz et al. (2019)
Cardiac	*Mhc*-Cre, *Dot1l* Exon 5–6 LoxP	H3K79me2/3 IF and IB of heart extract	Premature death (50% within 2 weeks post-birth). At P10: enlarged hearts, increased apoptosis, aberrant fetal cardiac gene expression. A maintenance of proliferating cells at P1 and P5. Downregulation of dystrophin and its exogenous expression post-birth rescued some of the KO phenotypes. Nguyen et al. (2011)
Endothelial cells	*Tie2*-Cre, *Dot1l* Exon 5–6 LoxP		Normal development at E12.5. E13.5: edema, hemorrhage spots, and lethality. The pups that were born died by 3 weeks and had chylous ascites. Yoo et al. (2020)
Lymphatic endothelial cells	*Lyve1*-Cre, *Dot1l* Exon 5–6 LoxP		No observable phenotype. Yoo et al. (2020)
Definitive hematopoietic stem cells	*Vav*-Cre, *Dot1l* Exon5 LoxP	H3K79me2 IB of peripheral blood nucleated cells	Varying degrees of anemia at 3–6 weeks. Decreased bone marrow cellularity. Reduced red blood cells and a smaller reduction of white blood cells. Bernt et al. (2011)
T cells	*Lck*-Cre, *Dot1l* Exon2 LoxP	H3K79me2 IHC and flow cytometry of thymus	Differentiation of naïve CD8^+^ to memory T cells in the absence of immunological challenge. De-repression of developmental genes and poor immune response. Kwesi-Maliepaard et al. (2020)
T cells	*Cd4-*Cre, *Dot1l* Exon2 LoxP	H3K79me2 IB of T cell subsets	Reduction in CD4^+^ T cells by cell death. Increased T cell receptor signaling with overproduction of IFN-γ, Reduced Th2/increased Th1 immune response. Scheer et al. (2020)
B cells	*Mb1*-Cre, *Dot1l* Exon2 LoxP	I. H3K79me2 flow cytometry in bone marrow and splenic B cells	I. 1.6-fold reduction in bone marrow B lineage cells by inhibiting pro-B to pre-B cell maturation at 6–8 weeks old. Inability to form germinal centers. Premature acquisition of plasma cell characteristics. Aslam et al. (2021) II. Reduction of peripheral mature B cells. Kealy et al. (2020)
Mature B cells	*Cd23*-Cre, *Dot1l* Exon2 LoxP	H3K79me2 IB of B cell subsets	Lack of germinal centers and memory B cells; reduced class-switched plasma cells upon challenge. Similar proliferation of B cells. Kealy et al. (2020)
Kidney connecting tubule/collecting duct	*Aqp2*-Cre, *Dot1l* Exon5 LoxP		Normal at 5 months, kidney fibrosis at 14 months. Wu et al. (2013); Zhang et al. (2020)

IF, immunofluorescence; IB, immunoblot; IHC, immunohistochemistry.

## Conclusions and Future Directions

Increased developmental potential is correlated with reduced DOT1L activity (Figure 2). In the 2C stage of development, DOT1L is evicted from the nucleus when pericentric heterochromatin is undergoing massive reorganization and transcription is activated from the zygotic genome. *In vitro*, ESCs are uniquely tolerant of DOT1L loss, and its catalytic inhibition greatly enhances reprogramming to pluripotency without causal transcriptional alterations. Reduction of H3K79me2 allows for the spread of the repressive modification H3K27me3 at select genes, and the increase of the activating modification H3K9ac at telomeres suggesting a context and gene-specific cross-talk of the DOT1L-network. The recent expansion of mouse models has revealed that DOT1L is broadly critical for specification and tissue organization. DOT1L is not required until organogenesis across species, far beyond the acquisition of H3K79me in the blastocyst. Organogenesis is a unique stage where pluripotent genes are fully shut off and transcripts diversify. Future studies of DOT1L-KO models at the resolution of single cells could uncover the role of this mysterious protein in development. The function of H3K79me in development and lineage specification is likely to be multifaceted and can be explored in multiple contexts as detailed below:

1) H3K79me2/3 are rapidly lost post-fertilization in the absence of DNA replication. The current school of thought is that H3K79me is diluted by nucleosome turnover (De Vos et al., 2011; Chory et al., 2019) but a potential demethylase has been reported in leukemia cell lines (Kang et al., 2018). Is H3K79me2/3 evicted by histone exchange, or is there an active H3K79 demethylase during development? If so, is this demethylase specific to the embryo?
2) Reduced H3K79me is correlated with increased developmental potential (Figure 2), but is it required? *In vitro*, would increased DOT1L activity prevent cycling to the 2CLC?
3) H3K79me3 is found at pericentric heterochromatin in fibroblasts, oocytes (Ooga et al., 2008), and ESCs (Yang et al., 2022). It is also localized on silent imprinted genes (Singh et al., 2010, 2011). Does H3K79me3 have a repressive function separate from H3K79me2 or does it accumulate at these silent regions because of low nucleosome turnover?
4) At the totipotent 2C stage, DOT1L is shuttled to the cytoplasm and enforced nuclear maintenance precipitates reorganization of pericentric heterochromatin to chromocenters. Does DOT1L have distinct functions throughout development and in different lineages?
5) Does H3K79me2 function as a book-keeping epigenetic modification that denotes genes for expression rather than having a specific activating role? If true, nuclear DOT1L at the 2C stage could methylate the major satellite repeats before they are protected within the chromocenter, establishing an aberrant pattern of expression.
6) H3K79me2 is enriched on the bodies of transcribed genes (Steger et al., 2008) and its levels correspond to activity (Wang et al., 2008; Veloso et al., 2014); yet disruption of DOT1L activity largely increases the expression of lowly expressed genes without H3K79me2 modification (Figure 3). Is this an artifact of sequencing or are many genes indirectly regulated by DOT1L? Could multiple published datasets of expression-matched H3K79me low vs. high genes be harnessed to understand the role of DOT1L in transcription across cell types?
7) In leukemias, DOT1L activity was shown to be required for maintenance, but not activation, of gene expression (Okuda et al., 2017). Is this because H3K79me2 enhances gene expression at DOT1L-tethered oncogenes (Mohan et al., 2010b)? Alternatively H3K79me2 was suggested to oppose H3K27me3 (Deshpande et al., 2014) and H3K9me3 *via* SIRT1/SUV39H1 (Chen et al., 2015) in leukemia. Is sustained gene expression due to an H3K79me2 epigenetic network, and are these networks active in non-transformed cell types?
8) DOT1L is only known to target H3K79 for methylation. Does DOT1L have additional histone or non-histone targets similar to other epigenetic modifiers?
